# Machine learning-based optimal crop selection system in smart agriculture

**DOI:** 10.1038/s41598-023-42356-y

**Published:** 2023-09-25

**Authors:** Sita Rani, Amit Kumar Mishra, Aman Kataria, Saurav Mallik, Hong Qin

**Affiliations:** 1 Department of Computer Science and Engineering, Guru Nanak Dev Engineering College, Ludhiana, Punjab 141006 India; 2 Department of Computer Science and Engineering, JAIN (Deemed-to-be University), Bengaluru, 562112 India; 3 Amity Institute of Defence Technology, Amity University Noida Campus, Sector -125, Noida, UP 201313 India; 4 Department of Environmental Health, Harvard T H Chan School of Public Health, Boston, MA 02149 USA; 5 Present Address: Department of Computer Science and Engineering, University of Tennessee, Chattanooga, TN 37403 USA

**Keywords:** Plant sciences, Environmental sciences, Environmental social sciences

## Abstract

The cultivation of most crops depends upon the regional weather conditions. So, the analysis of the agro-climatic conditions of a zone contributes significantly to deciding the right crop for the right land in the right season to obtain a better yield. Machine learning algorithms facilitate this process to a great extent for better results. In this paper, the authors proposed an ML-based crop selection model based on the weather conditions and soil parameters, collectively. Weather analysis is done using LSTM RNN and the process of crop selection is completed using Random Forest Classifier. This model gives better results for weather prediction in comparison to ANN. With LSTM RNN, the RMSE observed in Min. Temp. prediction is 5.023%, Max. Temp. Prediction is 7.28%, and Rainfall Prediction is 8.24%. In the second phase, the Random Forest Classifier showed 97.235% accuracy for crop selection, 96.437% accuracy in predicting resource dependency, and 97.647 accuracies in giving the appropriate sowing time for the crop. The model construction time taken with a random forest classifier using mentioned data size is 5.34 s. The authors also suggested the future research direction to further improve this work.

## Introduction

Due to the rapid population growth, the food requirement is increasing sharply which requires increased crop production. Cultivating suitable crops considering the soil, weather, and other parameters, can address this challenge^1–3^, which comes under the category of precision agriculture^4^. In the past few years, different data mining techniques have been used for making decisions in crop cultivation considering various environmental, soil, and other parameters. The most frequently used techniques can further be classified as statistical models and machine learning (ML) algorithms. In statistical methods, the organization of data is known in advance. On the other side, ML algorithms learn from existing data, known as 'dataset', and improve over time without explicit programming. In ML, data is termed training and testing data where training data is utilized to train the model, and test data is used to make predictions. ML algorithms are further classified as supervised and unsupervised, depending upon the type of data they operate on. In supervised ML, the training data is pre-labeled and used to make predictions. Whereas, in unsupervised ML, the training data is not labeled and this technique is used to understand the relationship among data.

**Figure 1:**
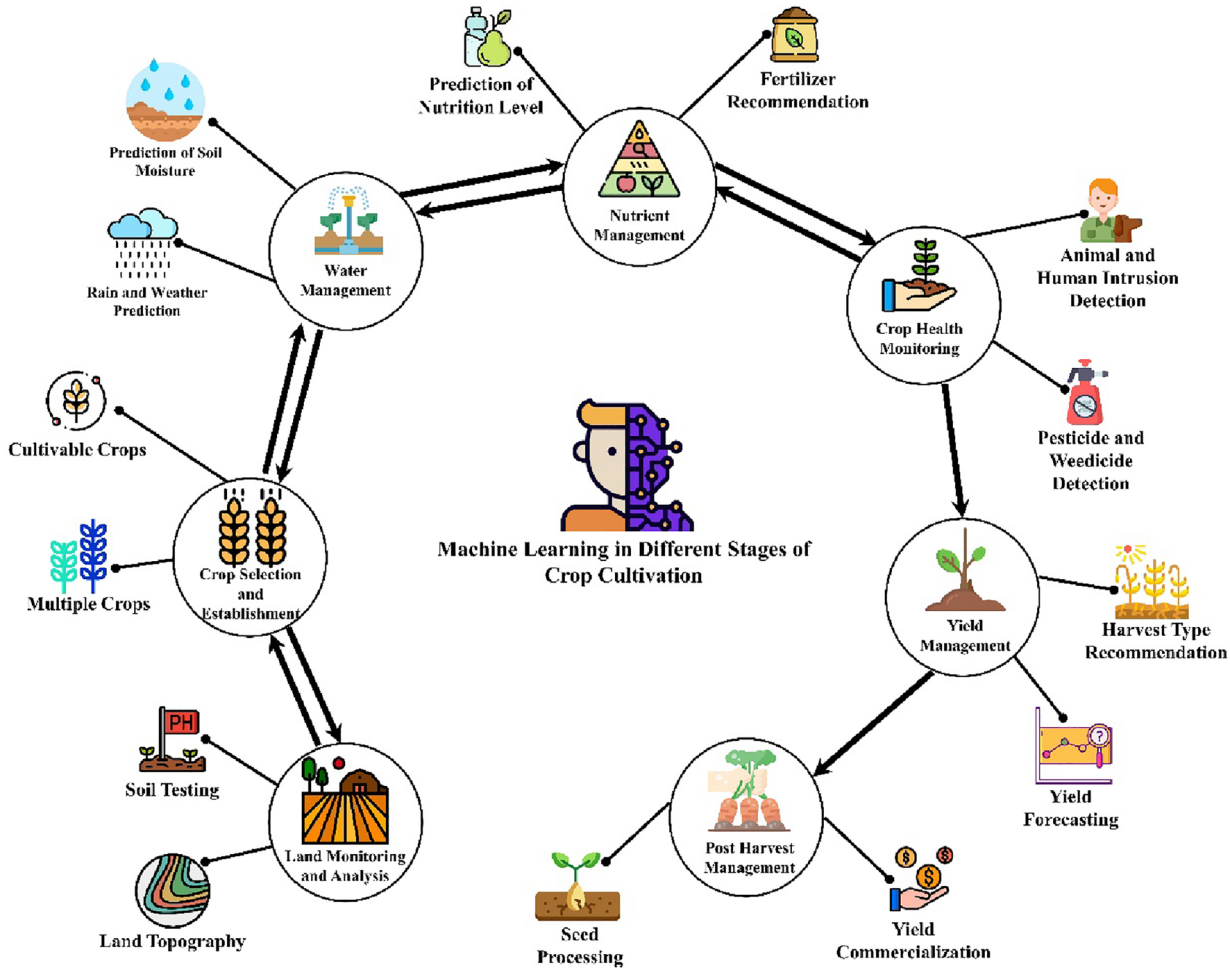
Machine learning in agriculture.

In the past, various ML techniques have been used to address the various challenges in the domain of agriculture, e.g., weather prediction^5^, plant disease classification^6,7^, intelligent irrigation^8^, yield prediction^9^, crop selection, etc.^10^, shown in Fig. 1.

In^11^, the authors presented a machine learning model to predict the suitable time to sow the crops by analyzing the weather conditions. The various ML techniques used in the proposed model are decision tree, support vector machine (SVM), and K-nearest neighbor (KNN). In ^12^, the authors proposed another ML-based model to estimate the crop's water requirement using adaptive neuro-fuzzy inference system (ANSIF) and partial least-square regression (PLSR) methods. This system can forecast the water requirement for a crop for one week. In^13^, Patil et al. proposed an automated ML system for disease diagnosis in grape plants. In this model, the authors have taken three parameters, i.e., moisture, humidity, and wetness of the leaves, and used Hidden Markov Model (HMM). In^14^, the authors reviewed the usage of ML algorithms for estimating nitrogen levels and predicting yield. In another paper, Dimitriadis presented an ML-based crop management model to analyze plant health and its water requirement^15^. Finally, in^16^, the authors presented a system to study the effect of environmental change on crop yield. It has been observed that weather conditions play a very significant role in many agriculture activities—likewise, pest management, water requirement, nutrient requirement analysis, yield prediction, etc. In^17^, the authors applied different ML techniques: artificial neural networks (ANN), SVM, decision tree, and naïve Bayes' for weather prediction. In^18^, the authors presented a recurrent neural network (RNN) model for weather forecasting. It has been observed from the literature that ML is playing a very significant role in different agricultural processes and various stages of crop cultivation.

Many parameters contribute to improving the production rate of a crop. These parameters are based on the environmental conditions and soil health, e.g., weather conditions, geographical area, soil type, nutrient level, pH value, etc.^19^. So, it is highly desired to use the consolidation of these parameters for selecting appropriate crops for land to obtain the maximum yield. To the best of the authors' knowledge, none of the existing models is using both parameters together. To that purpose, the fundamental objectives of this paper are:

- To study the existing ML models used for crop selection.
- To propose a new ML-based crop selection model considering both weather and soil parameters together.
- To analyze the performance of the proposed model.

In this paper, the authors have considered the soil parameters and climatic conditions of the state of Telangana for appropriate crop selection. But the proposed model can be mapped to the weather and soil conditions of any of the areas/zones.

In the paper's organization, Section “Literature survey” focuses on the previous work done in the area of suitable crop selection using various methods. Section “Proposed work” discusses the methodology used in this manuscript to select the suitable crop based on the weather conditions and soil parameters. Then, the results obtained from the proposed model are presented in Section “Experimental results”. Finally, in Section “Conclusions and future scope”, the paper is concluded by deriving the inferences and specifying the future directions.

## Literature survey

In agriculture, a lot of work is carried out to predict crop yield, select suitable crops based on weather conditions and soil data, etc. In^20^, the authors proposed a stochastic model for forecasting seasonal weather conditions. The proposed model conflates with another agriculture model developed for crop simulations to forecast crop produce and monetary return. This model was also fruitful in analyzing the risks associated with the various climatic conditions. The model was tested with the dataset of the crop Maize for two different geographical areas of the state of Argentina. In^21^, the authors proposed another aggregate model to manage various agricultural activities and predict the crop yield in Europe. The proposed model has been experimented with for the crop of wheat. The authors claimed that their model is more accurate and reliable than existing systems. This model was based on the probability distribution function. In^22^, the authors presented a yield prediction system for the state of Visakhapatnam. This model works in two layers. In the first phase, it uses a modular artificial neural network (MANN) to predict the rainfall in the region. In the second phase, the result of the first phase, along with some other parameters, has been used to predict crop yield. The authors implemented the model for the crops of bajra, ragi, rice, and maze. The authors present a nutrient-based soil classification system. This system divides the soil into three classes using KNN. This model used different soil parameters: nitrogen level, pH value, potassium, organic carbon, phosphorus, and micronutrients. In^23^, the authors presented a model to select the suitable crop to obtain the maximum yield. This model also recommends the order in which crops should be grown during the year on a piece of land to give maximum yield. The authors categorized the crops as seasonal, whole-year, short-term, and long-term. The parameters considered are water level to decide the suitability of the crop to be sown, soil type, type of crop, and weather conditions. This model advises crop sequence to obtain maximum produce based on crop type and sowing time. A smart crop selection model is presented in^24^. Authors used IoT to gather soil parameters and weather conditions in their system. Various soil parameters used to train the model are salinity, moisture, humidity, and temperature. Then 3D clustering was applied to compare the various farming techniques used for a particular crop. Several prediction algorithms such as random forest, naïve Bayes', and decision trees are presented by different authors to choose appropriate crops using weather conditions and soil parameters. In^25^, the authors presented another aggregate method to choose suitable crops considering weather and soil conditions. This model uses k-nearest neighbor, naïve Bayes', random forest, and CHAID algorithms. Classification criteria used in this model are soil color, root depth, texture, and drainage capacity of the land. In^26^, the authors presented a naïve Bayes' classification model to predict suitable crops considering various environmental parameters like temperature, rainfall, moisture, and pressure. Experiments were performed for rice, cotton, chilli, and maize crops. This model also suggests an appropriate time to sow and harvest a crop. In^27^, the authors highlighted the importance of agriculture and the dependency of the people on it in India. The authors discussed important soil parameters, like pH value, nutrition level, and drainage capacity which play an important role in crop selection and yield. Three different ML techniques, i.e., KNN, Decision Tree, and Naïve Bayes algorithm are applied by the authors for crop yield prediction. In^28^, authors used GBDT and ridge regression, KNN, and SVM to predict crop yield in Europe. Authors used soil parameters in their work to make yield predictions in the various regions of the country taking 35 different cases.

The literature shows that much work is carried out for suitable crop selection in the past, summarized in Table 1. But it has been observed that none of the previously proposed models considers both weather and soil parameters together for optimal crop selection. In this article, the authors proposed an ML-based optimal crop selection system that considers weather conditions, soil parameters, and cross-classification techniques to advise appropriate crops for a piece of land. It also suggests the most suitable time to sow the crop and required agricultural amenities which are not discussed in any previous papers.

**Table 1:** Summary of the ML algorithms/techniques applied in the literature for optimal crop selection.

References	Techniques used	Parameters	Strengths	Limitations
Apipattanavis et al.^20^	DSSAT	Weather	Seasonal production Risks are considered during the prediction	Inadequate spatial resolution
Cantelaube and Terres^21^	PDF	Weather	Suitable for weather-dependent decision making	Economic aspects are not covered
Khosla et al.^22^	ANN Support Vector Regression	Rainfall	Improved performance	Used climatic parameters only
Paul et al.^29^	Naive Bayes KNN	Soil	Guide for better crop production	Smaller Dataset
Kumar et al.^23^	ANN SVM	Soil	Gives yield prediction also	The prediction technique is not very efficient
Tseng et al.^24^	3D Cluster Analysis	Weather	The process of data cleaning is optimized	Environment management is not considered
Pudumalar et al.^25^	Random Forest KNN Naïve Bayes	Soil	Crop selection helps in yield maximization	A better prediction method needs to be used
Priya et al.^26^	Naïve Bayes	Soil	Promotes precision agriculture	Pesticide recommendations and irrigation needs are not provided
Malik et al.^27^	KNN Naïve Bayes Decision Tree	Soil	Comparative study of soil characteristics	Dataset is limited
Paudel et al.^28^	Ridge Regression KNN SVM GBDT Regression	Soil	Different spatial levels are also considered	Does not perform well during extreme years

## Proposed work

The authors divided the proposed model into two stages, i.e., phase 1 is deployed for weather prediction, whereas phase 2 is used for identifying the optimal crop. In this work, phase 1 is implemented using Recurrent Neural Networks (RNN) and in phase 2, Random Forest Classification is used for crop selection using predicted weather and soil parameters of the region. As the weather of any area for a day is also influenced by the weather conditions of the past day/s; so, RNN is observed as the most suitable ML algorithm for weather prediction in this work. In RNN, the output of a few layers act as input to the previous layer which eases the mapping of the problem of weather prediction to this algorithm. In this work, the authors used LSTM RNN due to weather dependencies on previous conditions. For appropriate crop selection, multiple weather and soil parameters are considered in this work which requires the combination of multiple decision trees to be considered, and in such cases random forest classifier can play a significant role in the problem domain.

**Figure 2:**
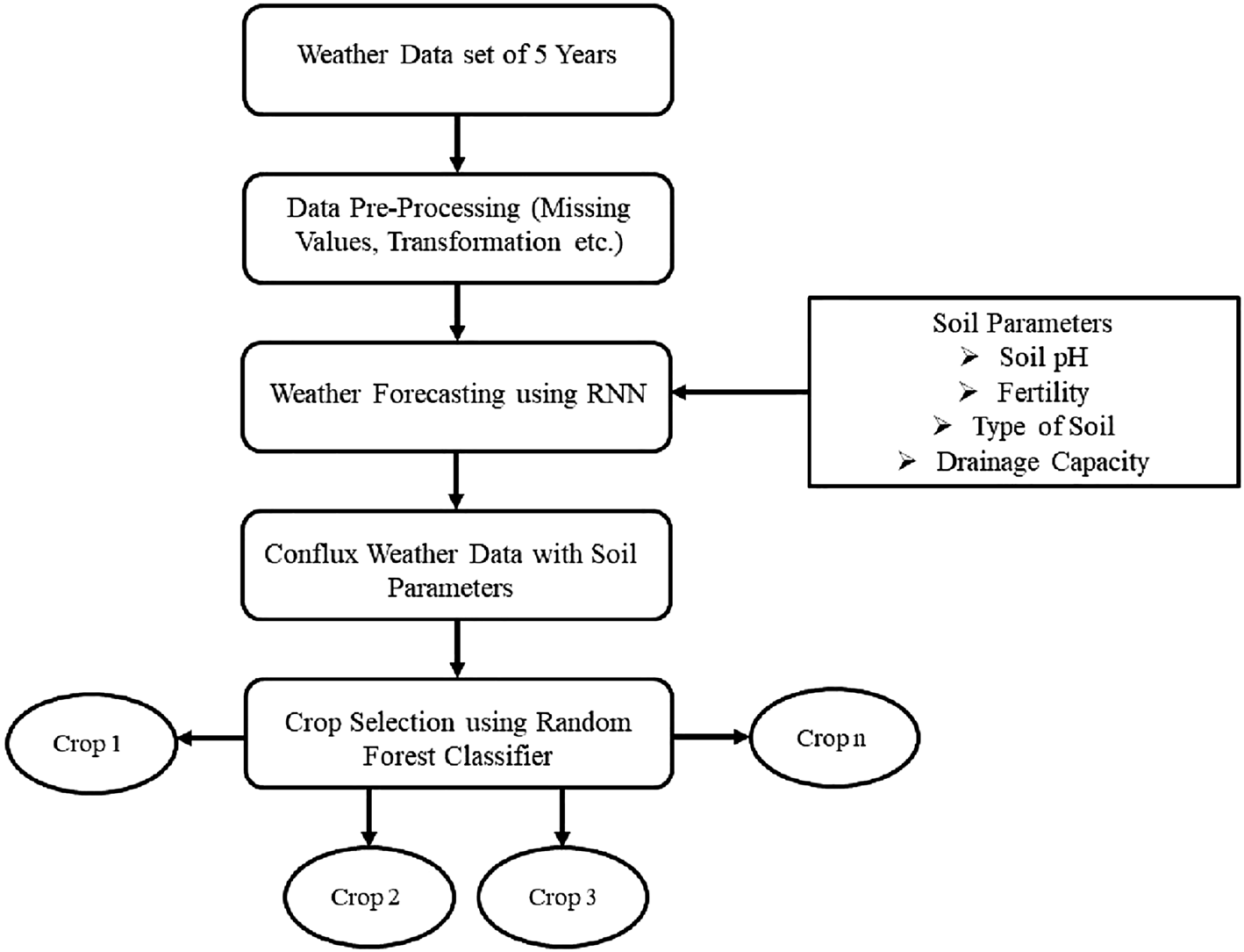
Flow chart: Proposed methodology.

The flowchart for the proposed work is presented in Fig. 2.

### Weather prediction

**Figure 3:**
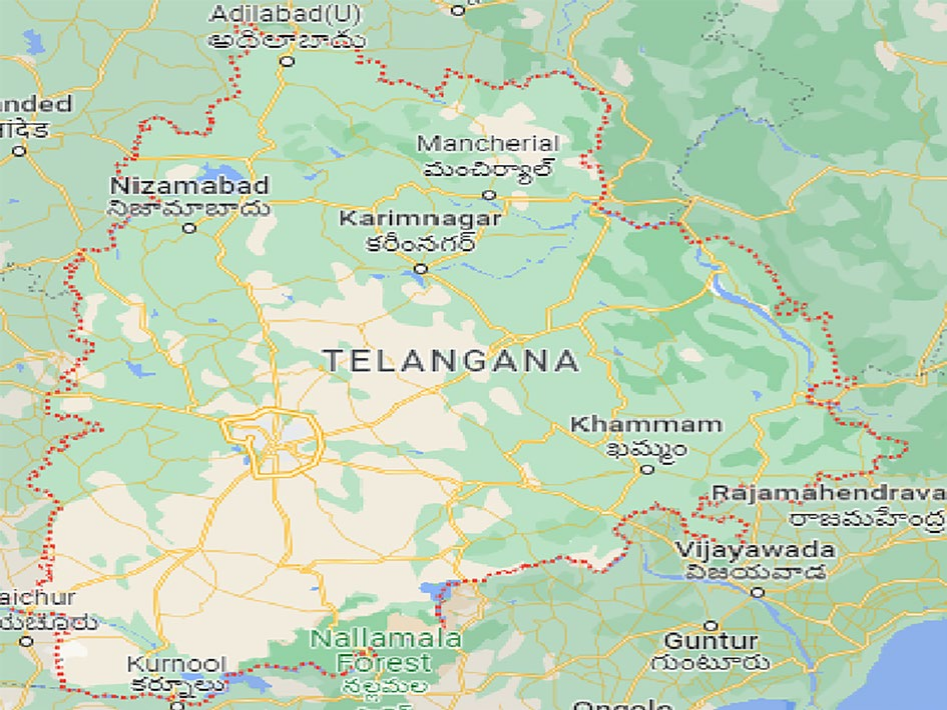
Telangana state of India: Area considered for this study. (*Source* Google Maps https://www.google.co.in/maps).

For this work, the authors considered the Telangana state of India, shown in Fig. 3. For weather prediction, data were collected personally from the data center of the National Remote Sensing Agency (NRSA), Hyderabad for the years 2015–2020.

The dataset comprised of approximately 1993 records. The features of the data were temperature, wind speed and its direction, humidity, sun hours, etc. The units of temperature, windspeed, and rainfall were Celsius, km/hour, mm respectively. In the very first step, the authors pre-processed the data to fill in the missing values, transformation, and normalization. The missing values were handled using the linear interpolation technique. Afterward, the data were processed to obtain the minimum and maximum values of the temperature for each day from the respective features. Subsequently, the authors converted the dataset to a common scale using min–max normalization^30^. Data preprocessing is done using pandas, scikit-learn, and minmax scalar libraries of python.

**Figure 4:**
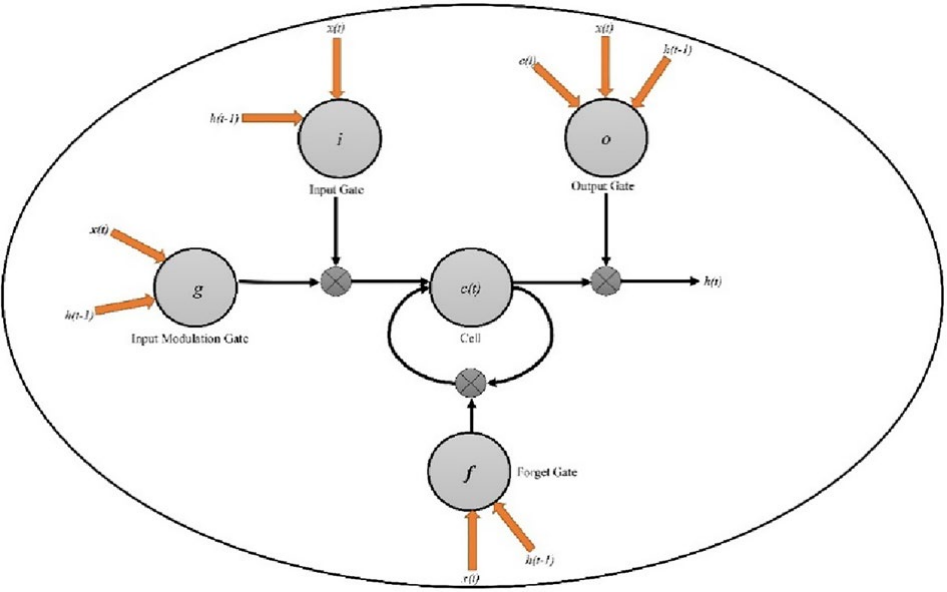
A single node of LSTM mesh.

For the processed data, RNN- an advancement of neural network (NN), is used to administer sequence dependence. In RNN, the input of the current step is taken from the output of the previous step, which aids the hidden state of RNN to remember the order. But, in conventional NN, every data element is taken as an independent entity. In the proposed work, long-short-term memory (LSTM) feedback-integrated RNN is used for the deployment of the model. A single node of the LSTM mesh is shown in Fig. 4.

Every LSTM node comprises three different gates, i.e., Input (I), Output (O), and Forget (F) gate. The revised input at present time-stamp T is presented by $G.$ The actual value of any of these gate's banks on the preceding hidden state ${H}_{I-1}$ and present input ${X}_{T}$, presented as below^31,32^.

1 $$I_{{T{ }}} = F{ }\left( {{ }W_{{I{ }}} X_{{T{ }}} + { }U_{I} { }H_{T - 1} + { }B_{F} } \right)$$

2 $$F_{{T{ }}} = F{ }\left( {{ }W_{F} X_{T} + { }U_{F} { }H_{T - 1} + { }B_{F} } \right)$$

3 $$G_{T} = \tanh \left( { W_{C} X_{T} + U_{C } H_{T - 1 } + B_{C } } \right)$$

The revised value at the node is determined as:

4 $$C_{{T{ }}} = { }I_{T} .G_{T} + { }F_{{T{ }}} .C_{T - 1}$$

The gate value is derived from the node state, preceding output, and present input to the node.

5 $$O_{T} = F{ }\left( {W_{{O{ }}} X_{T} + U_{{O{ }.{ }}} H_{T - 1} + { }V_{O} .{ }C_{T} + B_{O} } \right)$$

6 $$H_{T} = { }O_{T} .\tanh (C_{T} )$$

In this work, the authors have trained three RNN models, i.e., one for Min. Temp., one for Max. Temp., and one to predict the rainfall in the region. To predict the Max. Temp., the authors used the dataset for the years 2015–2018. Data for 2019–2020 is used to test and verify the model's accuracy. The data from the set is organized using vector as {X(1) …, X(K}3. For both types of forecasting, i.e., seasonal and 90 days prior, the dataset is mapped to an N X M matrix where in each row there is one input feature and 90 target values which are expressed as.

7 $$\left\{ {X\left( T \right),{ }X{ }\left( {T + 1} \right),{ }X\left( {T + 2} \right), \ldots X{ }\left( {T + 90} \right)} \right\}$$

**Figure 5:**
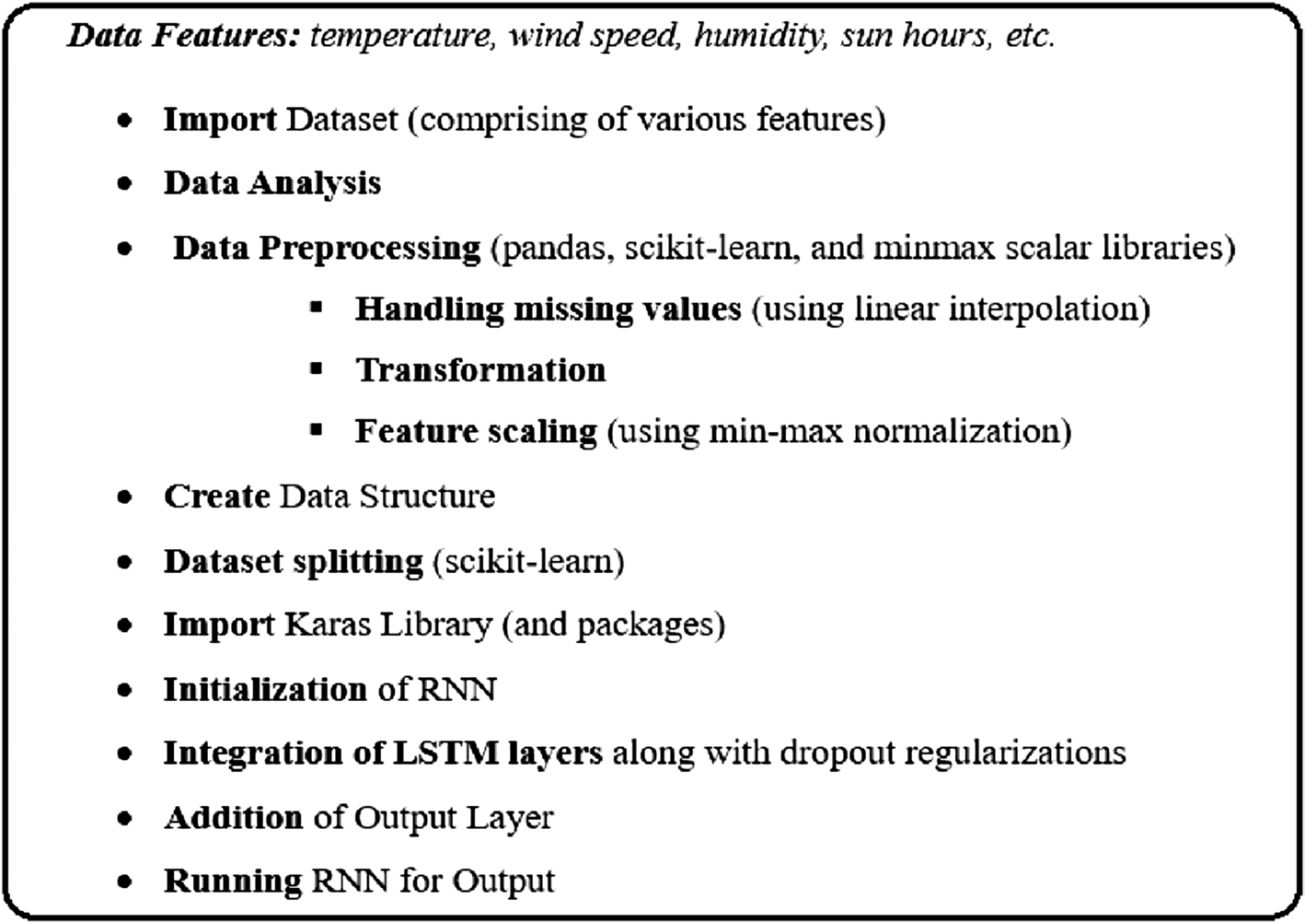
Pseudo code for weather prediction.

In the above discussion, K represents the size of the time series data. For example, k-90 represents that the dimension N and dimension M is 91 as the weather prediction is made by the LSTM, the model consists of an input layer, a hidden layer, and an output layer. The hidden layer is comprising 4 LSTM nodes. Min. Temperature and rainfall are also predicted similarly. The Pseudo code for weather prediction is shown in Fig. 5. Various data features from the data are fed to the input layer. The input layer processes the data and forwards it to the middle layer. The middle layer comprises many hidden layers. Each hidden layer has its own activation function, bias, and weight. Due to the dependency of weather conditions on past data, in this work, LSTM-RNN is used.

The results obtained using RNN are compared with ANN and found to be more accurate.

### Crop selection

As mentioned in Section “Weather prediction”, in this work, the Telangana state is considered for weather, soil, and crop data analysis. The geographical area of the state is classified into three agro-climatic belts, i.e., north Telangana, southern Telangana, and central Telangana. Different areas have different soil features. In north Telangana, the soil is red, shallow black, and profoundly calcareous. Southern Telangana comprises various textures of red soil, alluvial, and calcareous soil. Central Telangana is covered with red and calcareous soil^33^. The land is rated as low, medium, or highly fertile depending on its nutrient index. The main crops grown in the state are maize, rice, chilli, cotton, and soybean. Mainly these five crops are harvested over almost 5054 thousand hectares of the total agricultural land. The input to the crop selection model comprises soil and weather parameters. The various soil parameters for the algorithms are its type, pH value, water-preserving capability, and fertility. These soil and weather parameters predicted in the first phase, are collectively utilized to decide the appropriate crop for land. Before passing to the model, various categorical parameters, like type of soil, water capacity, fertility, etc. were encoded to the numerical values. The model can be utilized for both, i.e., seasonal and annual crop selection. For a season, the proposed model can recommend even more than one suitable crop and its requirements likewise water, and suggests an appropriate time to sow the crops. The example dataset utilized in the proposed model is presented in Table 2.

**Table 2:** Sample data to train the proposed ML model for the crop section.

Min. temp. (In Celsius)	Max. temp (In Celsius)	Rainfall (In mm)	Type of soil	pH Value	Water capacity	Fertility	Suitable crop
22	34	831	Black	6.5	Finely drained	Medium	Cotton
24	34	753	Loamy	6.0	Finely drained	High	Chilli
19	36	829	Calcareous	7.0	Finely drained	Low	Jawar
19	33	893	Alluvial	6.0	Finely drained	High	Rice
21	35	571	Loamy	7.0	Finely drained	Medium	Maize
23	32	551	Red	7.0	Finely drained	Medium	Castor
27	35	570	Alluvial	6.5	Finely drained	High	Soybean
35	40	857	Loamy	6.5	Finely drained	Medium	Red gram
30	40	340	Loamy	7.0	Finely drained	Medium	Sunflower
24	33	715	Black	7.5	Finely drained	High	Green gram

A total of 10 crops of the state of Telangana are considered in the proposed model. These crops are soybean, castor, green gram, sunflower, red gram, maize, chilli, cotton, jowar, and rice. But the model can be mapped for any number of crops and type of land. This work applies the random forest classification technique for reasonable crop prediction considering soil and water parameters. The random forest classifier uses a set of decision trees developed from a subset of training data. This classifier aggregates the output from each decision tree to decide the outcome. The class with the maximum number of votes is considered the outcome of the algorithm. The model is customized to check for the suitability of more than one crop for a particular land which is implemented using a threshold value. A crop is included in the list of appropriate crops if any decision tree using random forests presents the same crop as output with a value more than the threshold value.

8 $$Th = 2{*}\left( {\frac{Number\;of\;trees\;generated\;with\;Random\;Forest\;Classifier}{{Number\;of\;Classes}}} \right)$$

**Figure 6:**
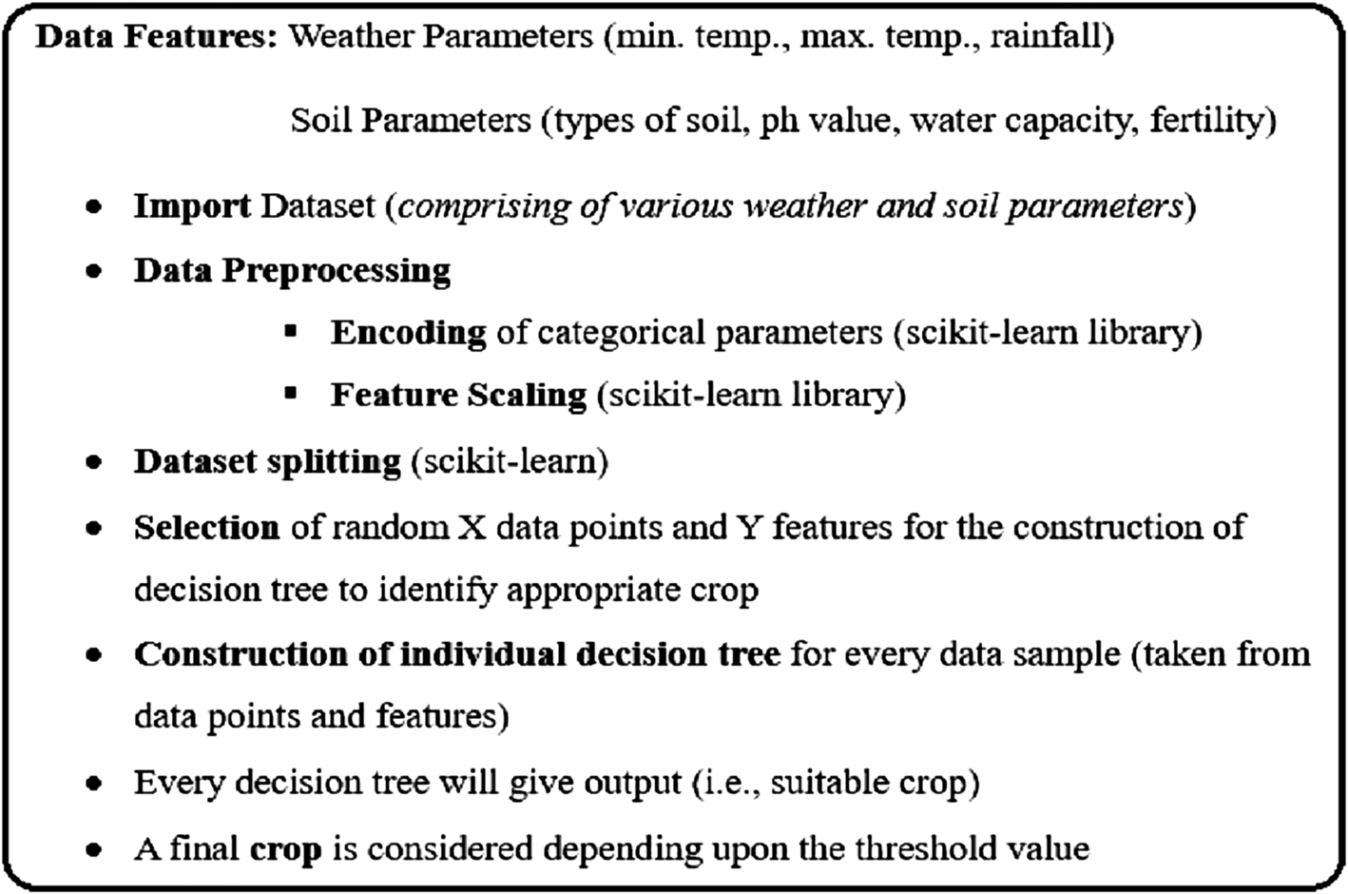
Pseudo code for crop selection.

The pseudocode for crop selection is shown in Fig. 6.

The output received with sample data using the classification algorithm is presented in Table 3.

**Table 3:** Performance comparison: RNN vs ANN.

ML technique	RMSE (%)		
	Min. temp	Max. temp	Rainfall
ANN	6.007	12.35	9.17
RNN	5.023	7.28	8.24

## Experimental results

**Figure 7:**
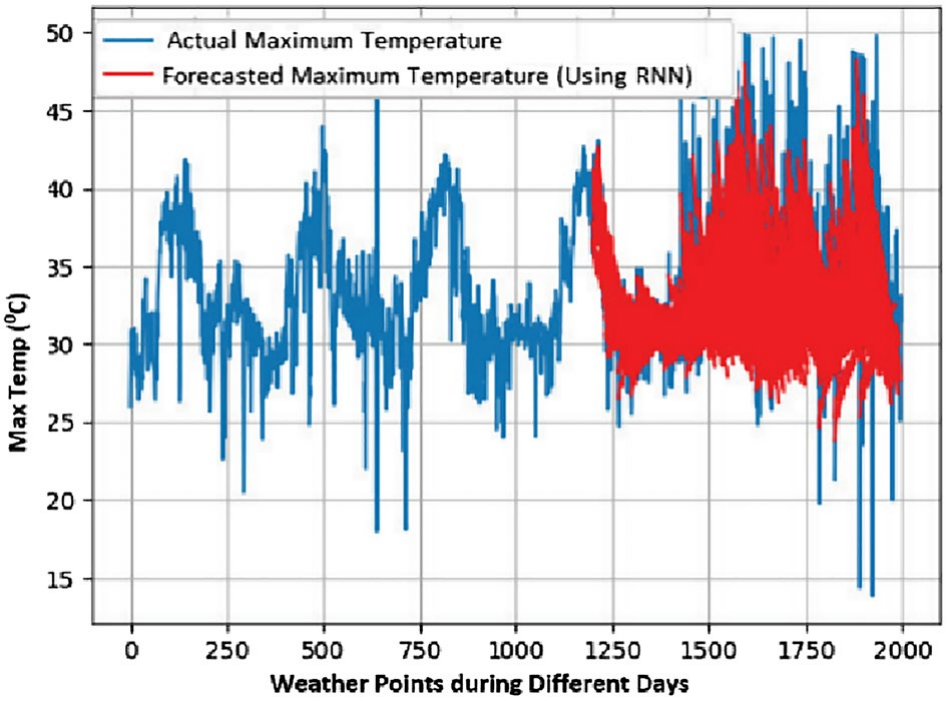
Max Temp prediction for 90 days.

**Figure 8:**
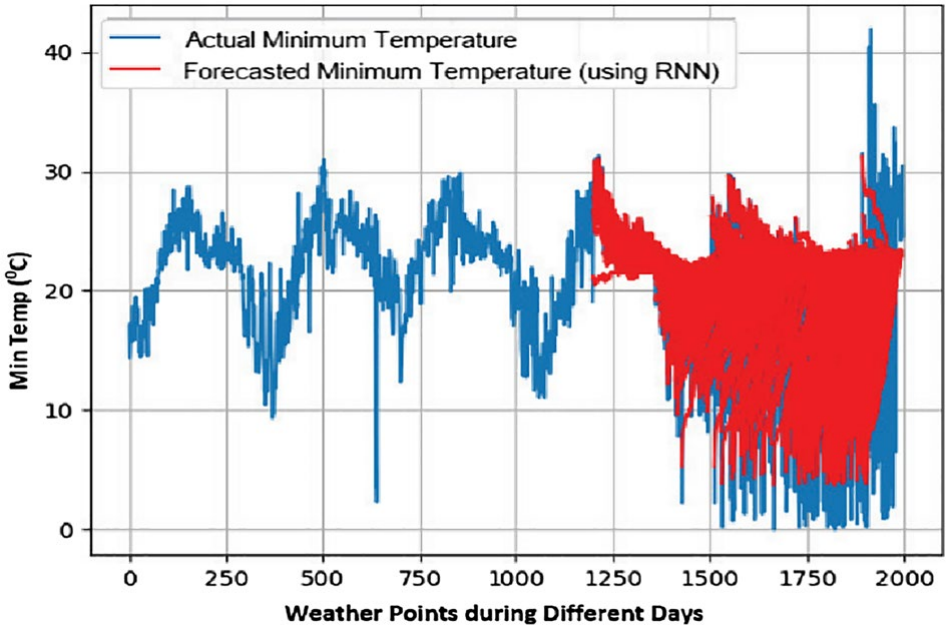
Min Temp prediction for 90 days.

**Figure 9:**
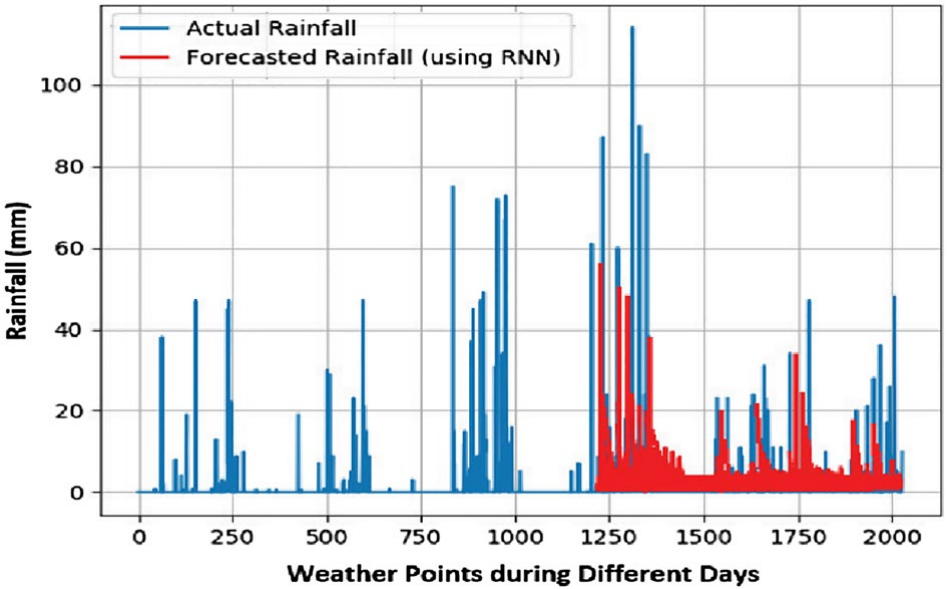
Rainfall prediction for 90 days.

In this work, a total of 1988 weather points are considered for a period of approximately six years, i.e., January 2015 to October 2020. A weather point is an entity showing meteorological information for a specific geographical point at a specific time. In this work, the weather point comprises a consolidation of temperature, wind speed, wind direction, humidity, and sun hours. From the used data, 60% of samples were used for the training of the model and the remaining 40% for the prediction. Three criteria for predicting appropriate seasonal crops are used, i.e., max temp, min temp, and degree of rainfall. Results for predicting these criteria are presented in Figs. 7, 8, and 9, respectively.

From the obtained results, it has been observed that RNN is more accurate in predicting time series with tolerable error. It is derived by comparing the results of RNN with ANN using the metric Root Mean Square Error (RMSE).

RMSE is one of the most often used methods for measuring prediction quality It uses Euclidean distance to demonstrate how far predictions differ from measured true values. Formally RMSE is defined as follows:

9 $${\text{RMSE}}\, = \,\sqrt {\mathop \sum \limits_{{{\text{i}} = 1}}^{{\text{n}}} \frac{{\left( {{\hat{\text{y}}}_{{\text{i}}} - {\text{y}}_{{\text{i}}} } \right)^{2} }}{{\text{n}}}}$$

where ${\widehat{\mathrm{y}}}_{1}$, ${\widehat{\mathrm{y}}}_{2}$, …, ${\widehat{\mathrm{y}}}_{\mathrm{n}}$ are predicted values, ${y}_{1}$, ${y}_{2}$, …, ${y}_{n}$ are observed values, and $n$ is the number of observations.

Lesser the value of RMSE for a model, the better the results. The results with both of these models are summarized in Table 3 and it can be easily analyzed that RNN has lesser value for RMSE for all the three parameters, i.e., Min. Temp., Max. Temp., and rainfall.

**Figure 10:**
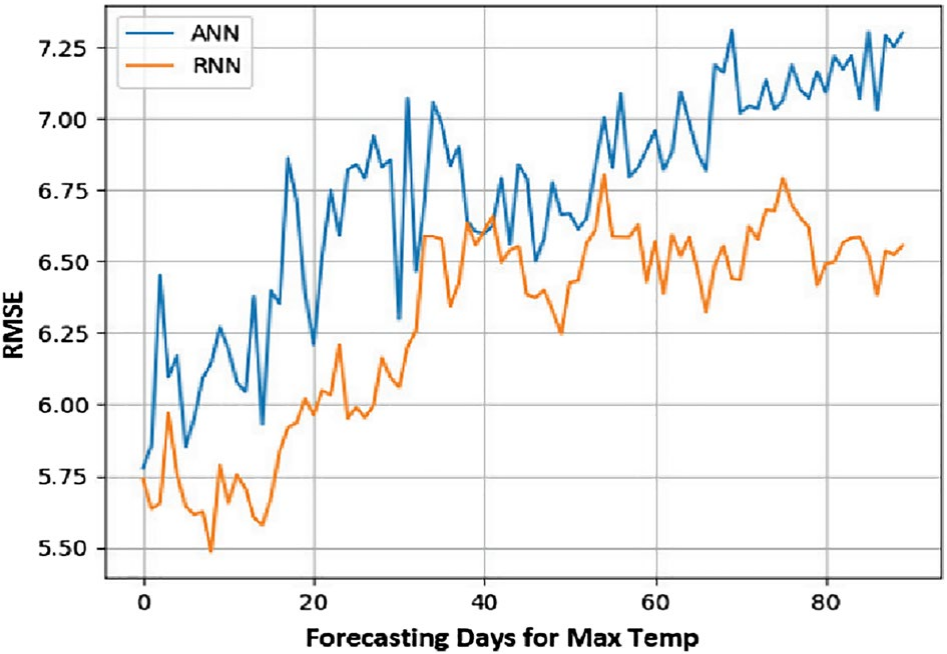
Max temp day ahead prediction: RMSE value.

**Figure 11:**
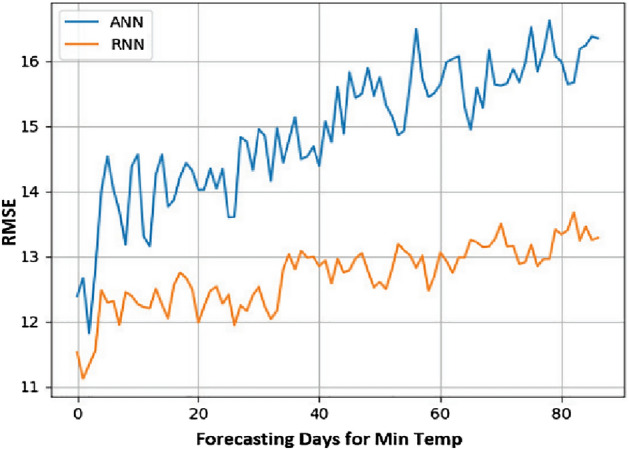
Min temp day ahead prediction: RMSE value.

**Figure 12:**
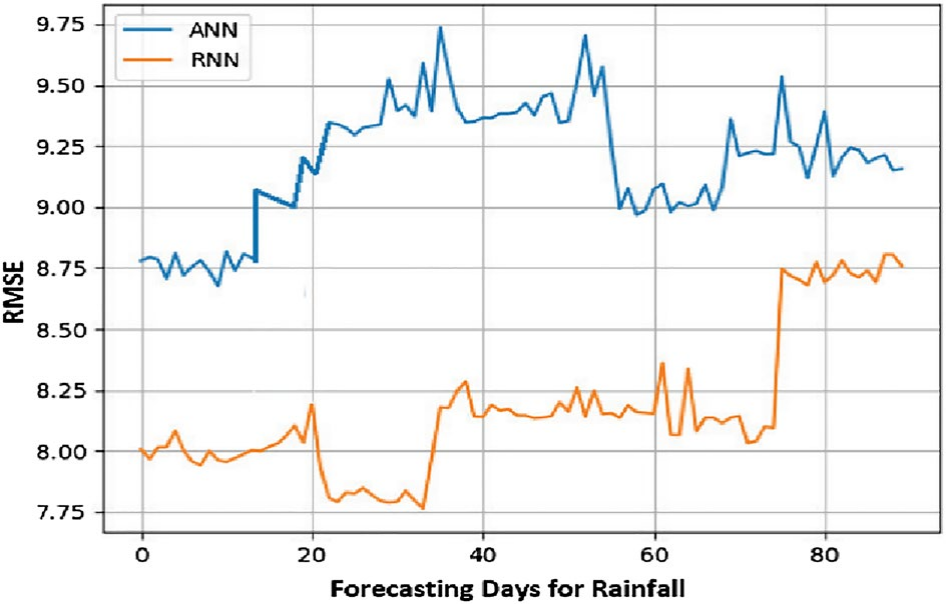
Rainfall prediction: RMSE value.

The comparison of RNN and ANN using RMSE for day-wise forecasting is also presented diagrammatically in Figs. 10, 11, and 12. It is imperative from the graphs that as the number of forecasting days increases, there is also an increase in prediction error for the day ahead prediction in all three cases.

Once the weather prediction is complete, the next step is to select a suitable crop depending on predicted weather conditions and soil parameters. In this work, the process of crop selection is implemented using the Random Forest Classification algorithm. Depending upon weather and soil parameters, the output obtained with the Random Forest classifier about the suitability of the crop for the season of Rabi is presented in Table 4.

**Table 4:** Suitability of the crop for the season of Rabi.

Selected appropriate crop	Dependency (Irrigation/Rainfall)	Appropriate sowing time
Maze	Irrigation	November-3rd week
Red gram	Rainfall	October-1st Week
Rice	Irrigation	December-3rd Week

Along with crop, the proposed method also suggests the sowing time and needed resources. From the data presented in Table 4, it is easily interpretable that some of the crops of the Rabi season require irrigation, whereas others can be grown with rainfall.

Using Random Forest Classier, observed accuracy is presented in Table 5. The performance of the used classifier is also compared with Decision Tree, and Random Forest Classifier is found better in performance. The model construction time taken with a random forest classifier using mentioned data size is 5.34 s.

**Table 5:** Performance comparison: Random Forest classifier and decision tree.

ML algorithm	Accuracy (%)		
	Crop selection	Dependency (Irrigation/Rainfall)	Appropriate sowing time
Random forest classifier	97.235	96.437	97.647
Decision tree	94.176	93.731	95.459

## Conclusions and future scope

ML is playing a significant role in the domain of agriculture. In this paper, the authors proposed an ML-based optimal crop selection model. In the proposed model, two primary criteria, i.e., weather conditions and soil parameters, are considered to select a suitable crop for a piece of land in a particular region. The model is trained using the weather and soil data from the state of Telangana. The process of weather forecasting is performed with LSTM RNN. The results obtained using RNN are also compared to ANN, and RNN is observed as a better prediction algorithm in this application domain. With RNN, the RMSE observed in Min. Temp. prediction is 5.023%, Max. Temp. prediction is 7.28%, and Rainfall Prediction is 8.24%. The selection of the suitable crop is made using Random Forest Classifier. This model suggests the suitable crop for land, its resource dependency, and appropriate sowing time. The performance of the Random Forest Classifier is compared with the Decision Tree classifier for all three outputs and observed better in performance. Random Forest Classifier showed 97.235% accuracy for crop selection, 96.437% accuracy in predicting resource dependency, and 97.647% accuracy in giving the appropriate sowing time for the crop. The model construction time taken with a random forest classifier using mentioned data size is 5.34 s.

Although, this work is carried out for the Telangana state of India, but can be mapped to any other geographical area. As per the authors’ knowledge, this is the only model which considers the consolidation of weather and soil parameters for optimal crop selection, i.e., the key novelty of this work. In this model, the model construction time for Random Forest Classifier is also given which is not done in any of the previous ML-based crop selection models.

But LSTM RNN technique, used in the first phase of this work, is limited by the size of the context window. LSTM only considers a limited number of inputs when making predictions; anything outside of the context window is ignored completely. There are two possible solutions to this limitation: train a larger LSTM with more cells which requires more weather data or utilize attention-based models. Consequently, using any of these methods can be adopted to improve the performance of the model to the next level. Random Forest Classifier, used in the second phase, requires more computational power and time as it builds numerous trees to combine their outputs. Due to the ensemble of decision trees, it also suffers interpretability and fails to determine the significance of each variable. Both of these limitations lead the new directions to the researchers for the future scope of this work. This model can be further improved by incorporating sensors to gather real-time more precise weather conditions and soil parameters specific to the farm, which will enhance the model's efficiency. Considering additional soil parameters like the degree of N-P-K nutrients and temperature can also help to obtain more accurate results.

## Data Availability

All data included in this study are available upon request.
